# Functional outcomes evaluation after radial head arthoplasty in DR. Syaiful Anwar General Hospital: Case series

**DOI:** 10.1016/j.ijscr.2020.04.004

**Published:** 2020-05-08

**Authors:** Agung Riyanto Budi Santoso, Thomas Erwin Christian Junus Huwae, Dedde Aditya Rachman, Marvin Anthony Putera

**Affiliations:** a Orthopaedic and Traumatology Department, Upper Extremity and Microsurgery Reconstruction Division, Saiful Anwar Hospital, Jalan Jaksa Agung Suprapto No. 2, Klojen, Kota Malang, Jawa Timur, 65112, Indonesia; b Orthopaedic and Traumatology Department, Saiful Anwar Hospital, Jalan Jaksa Agung Suprapto No. 2, Klojen, Kota Malang, Jawa Timur, 65112, Indonesia

**Keywords:** Functional outcomes, Radial head arthroplasty, Range of movement

## Abstract

**Introduction:**

Radial head arthroplasty (RHA) has become one of the mainstay surgical treatment for radial head fracture in certain conditions. Many previous studies compared the superiority of either open reduction and internal fixation (ORIF) and RHA with inconclusive result. Our case series aim to evaluate the functional outcome of the patients treated with RHA in our institution.

**Presentation of case:**

We evaluated three patients who had RHA in 2018 and 2019. The function of flexion, extension, supination and pronation of the elbow was evaluated. The range of motion (ROM) was assessed in both active and passive way.

**Discussion:**

An abundant of literatures stated that RHA result is superior than radial head resection and ORIF. Our case series present a satisfying result on pain and a fair result on functional outcome from the range of motion (ROM) evaluation on patient follow-up.

**Conclusion:**

A good follow up for post-operative care plays an important role in management of RHA to minimize the rate of complications. In this case series, the functional outcomes after RHA is good, but a larger number of patient and longer follow up duration is needed for a better analysis.

## Highlights

• Three case of radial head fracture treated with radial head arthroplasty.
• Evaluation of the functional outcomes after operative treatment of radial head arthroplasty.
• Functional outcomes assessment with active and passive range of motion.
• Satisfying result on pain and a fair result on functional outcome on patient follow-up.
• Good follow up is important to minimize the complications of radial head arthroplasty.

## 1 Introduction

Radial head fractures contribute to one-third of fractures around the elbow joint and 1.5–4% from all fractures. Fall on an outstretched hand in an active young patient has become the most common mechanism in this type of injury [1]. A complex injury pattern such as comminuted fractures of the radial head could have several mobile parts with no soft tissue envelope. Moreover, it often related to ligamentous and osseous injuries of the forearm or elbow. The goal of operative treatment for radial head fractures, whether with open reduction and internal fixation (ORIF) or acute prosthetic joint replacement, is to avoid subluxation or dislocation of the elbow joint by reconnecting the radio-humeral joint to achieve stability and joint alignment [2]. The most important structure which attach the elbow joint is the medial collateral ligament (MCL), which act as a primary constraint for valgus stress of the elbow. The MCL is also strengthened by radial head as a secondary stabilizer for valgus stability. Thus, radial head preservation is the utmost goal to avoid chronic instability in fractures involving ligament and soft tissue. Decreasing grip strength, cubitus valgus, proximal migration of radius and longitudinal instability, and ulnar neuropathy has been described by many researchers as a daunting complications of radial head resection. Because of that, many orthopaedic surgeons favor radial head arthroplasty as primary option of treatment in radial head fractures, ultimately for Mason type III and IV [3].

The aim of this case series is to evaluate the functional outcome of the patients after operative treatment of radial head arthroplasty.

## 2 Presentation of case

All of the surgeries was performed by the author under general anesthesia. The patient positioned supine for lateral access. The surgery performed through lateral Kocher/Kaplan approach to access the radial head. The radial head and neck resected and the neck trimmed to fit the prosthesis. The author inserts the chosen prosthesis which had been measured for its length and stability. Post-operative care protocol was delivered accordingly.

**Fig. 1:**
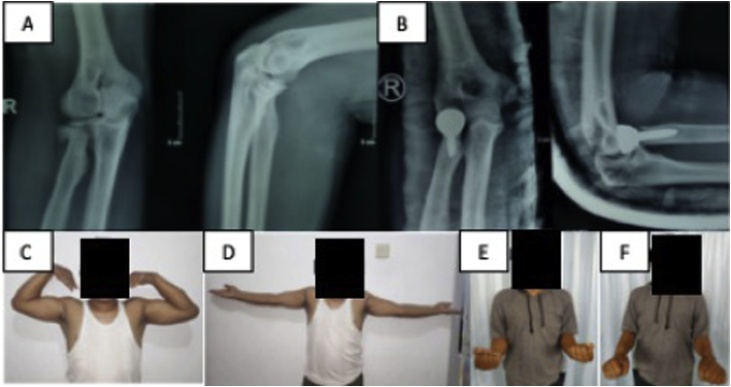
**A** Pre-operation AP/Lateral X-Ray of the right elbow from the first patient showing displacement and comminution of the radial head. Fig. 1**B** Post-operation AP/Lateral X-Ray of the right elbow from the same patient. The injury was treated with radial head arthroplasty. Fig. 1**C–F** Active ROM evaluation of the same patient; elbow flexion of 110°; elbow extension of 10°; elbow supination of 85°; elbow pronation of 75°.

We report three patients in this case series. The research work of this study has been reported in line with the PROCESS criteria [4]. The first patient is a 35 years old male, with surgery due to close fracture of right radial head after fall from 10 m height (Fig. 1A). Later on, the patient’s right elbow and left knee was operated in February 2018 (Fig. 1B).

We evaluated the post-operation functional outcome of all three patients who had radial head arthroplasty in 2018 and 2019. The function of flexion, extension, supination and pronation of the elbow was evaluated. The range of motion (ROM) was assessed in both active and passive. A year after the surgery, elbow joint ROM which had been operated was evaluated.

From the first patient, active ROM of elbow flexion was 110° (Fig. 1C), extension was 10° (Fig. 1D), supination was 85° (Fig. 1E), pronation was 75° (Fig. 1F), while passive ROM of elbow flexion was 125°, extension was 10°, supination was 85°, pronation was 75°.

**Fig. 2:**
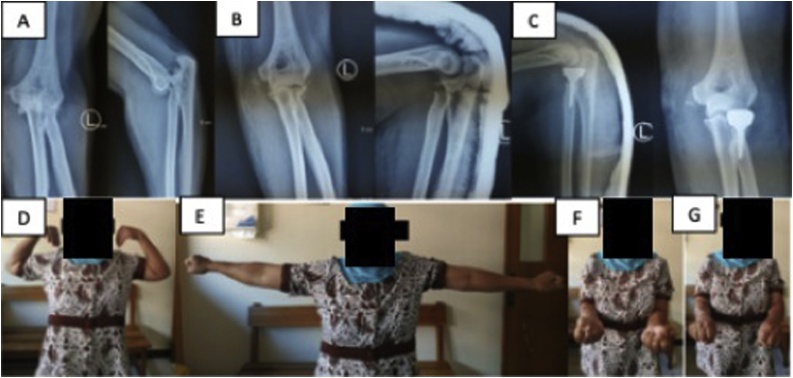
**A** Pre-operation AP/Lateral X-ray of the left elbow from the second patient before closed reduction was performed. The elbow was dislocated and the radial head was fractured. Thus, it is Mason type IV injury. Fig. 2**B** AP/Lateral X-ray of the left elbow from the same patient after closed reduction and application of posterior slab. Fig. 2**C** Post-operation AP/Lateral X-ray of the left elbow from the same patient. This patient was managed with prosthetic replacement of the radial head. Fig. 2**D–G** Active ROM evaluation of the same patient; elbow flexion of 140°; elbow extension of 5°; elbow supination of 80°; elbow pronation of 70°.

The second patient is a 53 years old female with close fracture dislocation of left elbow after fall on an outstretched hand in a volleyball game (Fig. 2A and B). Her left elbow was operated in December 2018 (Fig. 2C). From the second patient, active ROM of elbow flexion was 140° (Fig. 2D), extension was 5° (Fig. 2E), supination was 80°, pronation was 70°, while passive ROM of elbow flexion was 140°, extension was 0°, supination was 85° pronation was 75°.

**Fig. 3:**
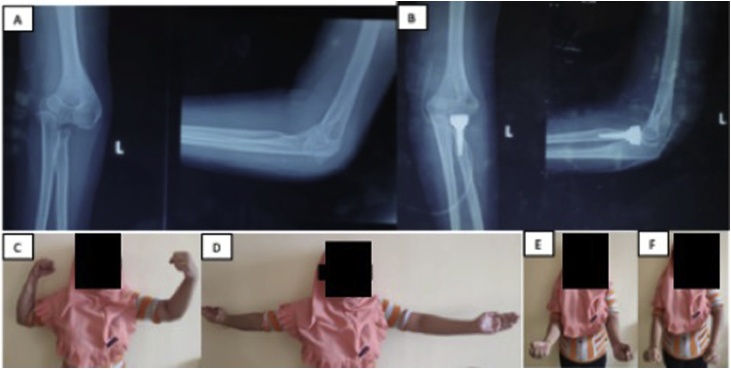
**A** Pre-operation AP/Lateral X-Ray of the left elbow from the third patient. The radial head was displaced >2 mm and conservative treatment would likely to fail, so the patient undergo surgery. Fig. 3**B** Post-operation AP/Lateral X-Ray of the left elbow from the same patient. The patient was also treated with radial head arthroplasty. Fig. 3**C–F** Active ROM evaluation of the same patient; elbow flexion of 80°; elbow extension of 10°; elbow supination of 80°; elbow pronation of 20°.

The third patient is a 38 years old female with neglected close fracture dislocation of the left elbow (terrible triad injury) (Fig. 3A). Patient had her elbow massaged, before operated in January 2019 (Fig. 3B).

From the third patient, active ROM of elbow flexion was 80° (Fig. 3C), extension was 10° (Fig. 3D), supination was 80° (Fig. 3E), pronation was 20° (Fig. 3F), while passive ROM of elbow flexion was 90°, extension was 5°, supination was 85°, pronation was 45°.

## 3 Discussion

Fractures of the radial head comprises about 5% of all fractures occurring in adults and about 15% of trauma to the elbow [2]. Radial head fractures are responsible for about one-third from all of elbow fractures. The incidence was estimated 2.5–2.8 per 10,000 inhabitants each year. The mean age of the patients sustaining this injury is relatively young and mainly in productive working individual of 44–47.9 years. Before the age of 50, the incidence in male patients is larger than female patients, but after the age of 50 years, the latter is more dominant. It is probably because of the presence of osteoporosis among the female individuals [5]. The most common etiology of radial head fractures is direct trauma. Static loading traversing the elbow was transmitted through the radio-capitellar articulation, bearing 60% of the elbow force [6].

In 1954 Mason observed the pattern of injuries in 100 patients with radial head fracture and classified them to 3 groups. Mason type I fractures of the radial head involved fractures with no displacement, type II fractures were displaced marginal sector fractures, and type III fractures were comminuted. Later in 1962 Johnston added the fourth classification which comprises dislocation of the elbow as a type IV to the system [5].

The principle treatment of radial head fracture was determined from its type of classification according to Mason. Type I injuries are treated non-surgically; Type II injuries could be managed conservatively or if displaced with ORIF; Type III injuries with ORIF or radial head arthroplasty. Resection of the radial head is indicated only for isolated radial head fractures without ligament injury [7].

Radial head arthroplasty (RHA) becoming a more popular option in recent years for the treatment of radial head fracture Mason type III and IV. Many studies state that RHA showed a superior result compared to radial head resection and open reduction and internal fixation, but some were against it, stating that the latter option is better in term of functional outcomes, number of revision surgery and post-operative early and late complications [1,3,4,7,8].

A review from Kaur et al., show that RHA has good to excellent functional outcomes in short to midterm follow-up. The postoperative outcomes of RHA varies to some certain factors such as patient-related, socioeconomic, education, work status, etc. Standardized report of complication and revision rate are needed to avoid an under-estimation of the failure rate [1].

Catellani et al. said that few papers recommend resection as a better option for isolated radial head fractures unassociated with ligaments injuries because of a lack of statistical clinical differences between RHA and radial head resection. No substantial differences in patient outcomes at medium and long-term follow-up [3]. A similar study from Akman et al. show that resection arthroplasty is an effective method for the treatment of isolated comminuted radial head fractures because of the less demanding technique [8]. All of our patient in this case series show fairly good functional outcomes even when some papers is saying that radial head resection is better. Slightly impaired ROM is occurred in the third patient, but this limitation does not affect her activity daily living significantly and the patient generally satisfied with the outcome of the surgery.

The limitation of our case series is the small number of patients due to short period of follow up. Patients were satisfied with the results on pain and functional outcomes regarding ROM were fair overall.

## 4 Conclusion

A good follow up for post-operative care plays an important role in management of RHA to minimize the complications. In this case series, the functional outcomes after RHA is fairly good, but a bigger number of patient and longer follow up is needed for a better analysis.

## Declaration of Competing Interest

None.

## Funding

The study does not use fund from any sponsors.

## Ethical approval

The study has been approved by the Ethical Committee of Medical Research Saiful Anwar State Hospital, Indonesia. This study has been in accordance with Declaration of Helsinki.

## Consent

Informed consent has been obtained from each patient.

## Registration of research studies

This study has been registered with code researchregistry5270.

## Guarantor

Agung Riyanto Budi Santoso.

## Provenance and peer review

Editorially reviewed, not externally peer-reviewed.

## CRediT authorship contribution statement

**Agung Riyanto Budi Santoso:** Conceptualization, Writing - original draft, Supervision. **Thomas Erwin Christian Junus Huwae:** Methodology, Data curation. **Dedde Aditya Rachman:** Software, Investigation, Conceptualization, Data curation. **Marvin Anthony Putera:** Writing - review & editing.
